# Ancient Function of Teneurins in Tissue Organization and Neuronal Guidance in the Nematode *Caenorhabditis elegans*

**DOI:** 10.3389/fnins.2019.00205

**Published:** 2019-03-08

**Authors:** Ulrike Topf, Krzysztof Drabikowski

**Affiliations:** Institute of Biochemistry and Biophysics, Polish Academy of Sciences, Warsaw, Poland

**Keywords:** teneurin, TEN-1, *C. elegans*, basement membrane, latrophilin, LAT-1, axon guidance

## Abstract

The nematode *Caenorhabditis elegans* expresses the *ten-1* gene that encodes teneurin. TEN-1 protein is expressed throughout the life of *C. elegans*. The loss of *ten-1* function results in embryonic and larval lethality, highlighting its importance for fundamental processes during development. TEN-1 is expressed in the epidermis and neurons. Defects in neuronal pathfinding and epidermal closure are characteristic of *ten-1* loss-of-function mutations. The molecular mechanisms of TEN-1 function in neurite outgrowth, neuronal pathfinding, and dendritic morphology in *C. elegans* are largely unknown. Its genetic redundancy with the extracellular matrix receptors integrin and dystroglycan and genetic interactions with several basement membrane components suggest a role for TEN-1 in the maintenance of basement membrane integrity, which is essential for neuronal guidance. Identification of the *lat-1* gene in *C. elegans*, which encodes latrophilin, as an interaction partner of *ten-1* provides further mechanistic insights into TEN-1 function in neuronal development. However, receptor-ligand interactions between LAT-1 and TEN-1 remain to be experimentally proven. The present review discusses the function of teneurin in *C. elegans*, with a focus on its involvement in the formation of receptor signaling complexes and neuronal networks.

## Introduction

Teneurins are large single-pass transmembrane glycoproteins that are conserved in most animals with a nervous system (Tucker, 2018). Teneurins were first discovered in *Drosophila* (Baumgartner and Chiquet-Ehrismann, 1993; Baumgartner et al., 1994) and later described in *Caenorhabditis elegans* (Drabikowski et al., 2005) and vertebrate models (Minet et al., 1999). Teneurins are involved in several developmental processes in invertebrate models and expressed most prominently in developing neuronal tissues, contributing to neuronal patterning and axon guidance (Drabikowski et al., 2005; Tucker et al., 2007; Silva et al., 2011; Mosca et al., 2012; Mosca, 2015). The family of teneurin proteins is characterized by a distinct protein domain architecture. Their extracellular domain consists of eight epidermal growth factor (EGF)-like repeats, a region of conserved cysteines, and unique tyrosine and aspartate (YD)-repeats and is highly conserved among vertebrates and invertebrates. The structures of some extracellular domains of chicken Ten2 and mouse Ten3 were recently solved, revealing a previously unpredicted TTR transthyretin-related domain that plays roles in protein aggregation and lipid recognition in other teneurin-unrelated proteins (Jackson et al., 2018; Li et al., 2018). Unlike the extracellular domain, the composition of the intracellular domain of teneurin proteins, with exception of some predicted phosphorylation sites, is very different between vertebrates and invertebrates. The intracellular domain can be cleaved off and translocate to the nucleus, whereas the extracellular domain can be released into the extracellular milieu. The ability of the intracellular domain to mediate cellular signaling within the nucleus was first observed in a cell culture model of vertebrate teneurin-2, in which overexpressed variants of teneurin-2 colocalized with promyelocytic leukemia protein (PML) bodies (Bagutti et al., 2003). However, the intracellular domain of the endogenous teneurin protein was found in the nucleus only in *C. elegans* (Drabikowski et al., 2005). Some studies have described a nuclear function of the teneurin intracellular domain that regulates transcription as a transcriptional repressor or activator (Bagutti et al., 2003; Nunes et al., 2005; Scholer et al., 2015; Glendining et al., 2017). However, the mechanism by which the membrane-spanning full-length teneurin protein is released to the intracellular domain from the plasma membrane is mostly unknown. Furin-cleavage sites between the transmembrane domain and the EGF-like repeats were suggested to be one such processing (Tucker and Chiquet-Ehrismann, 2006; Kenzelmann et al., 2007). This was supported by experiments in which recombinant avian teneurin-2 protein was cleaved by furin protease (Rubin et al., 1999). Similar to the mechanism of processing, signals that trigger the release of the intracellular domain remain to be discovered. Efforts to identify binding partners of the extracellular domain revealed various interactions that contributed to deciphering teneurin function as an organizer of neuronal networks (Mosca, 2015). Vertebrate teneurins form homo- and heterophilic interactions (Feng et al., 2002; Rubin et al., 2002; Beckmann et al., 2013). In *Drosophila*, teneurins mediate synaptic connections and neuromuscular connections via homophilic interactions (Hong et al., 2012; Mosca et al., 2012). In hippocampal neurons, teneurin-2 acts as a postsynaptic receptor for latrophilin (Silva et al., 2011). However, the ways in which this interaction contributes to synapse formation are unknown. Moreover, teneurin-1 interacts with beta-dystroglycan, resulting in cytoskeletal rearrangements (Chand et al., 2012). Whether teneurin-1 is expressed post- or presynaptically remains unclear. In *Drosophila*, an interaction between ten-m and integrin in motor neurons and muscles was proposed to be important for normal synaptic function, but the mechanism by which this occurs is unclear (Mosca et al., 2012; Dani et al., 2014). Studies in *C. elegans* revealed a fundamental role for teneurin in tissue organization and neuronal network development and maintenance (Drabikowski et al., 2005; Trzebiatowska et al., 2008; Morck et al., 2010; Topf and Chiquet-Ehrismann, 2011; Promel et al., 2012).

The present mini review provides an overview of the various identified genetic interactions with the *ten-1* gene in *C. elegans*, providing insights into its ancient function. We focus especially on *ten-1*-latrophilin connections, which are discussed within the context of recent findings in vertebrate models.

## Ten-1 Expression and Loss-Of-Function Phenotype

Most species express several teneurin paralogs (Tucker et al., 2012). Genetic redundancy has impeded investigations of their biological functions because single deletions show minimal phenotypic alterations. In contrast, the *C. elegans* genome encodes only one teneurin gene, *ten-1*. This fact, combined with the tremendous genetic tractability of the model organism, makes *C. elegans* an attractive system to investigate the biological significance of teneurins. Expression of the *ten-1* gene is under the control of two promoters that give rise to two transcript versions. These transcripts only differ in the length of the part that codes for the intracellular domain and thus were named short *ten-1a* and long *ten-1b*. These two forms of TEN-1 are expressed throughout worm development and in many tissues but have distinct expression patterns. An extensive analysis of TEN-1 expression was performed, in which green fluorescent protein (GFP) was expressed under two different promoters, p*ten-1a* (which controls the expression of TEN-1L) and p*ten-1b* (which controls the expression of TEN-1S). p*ten-1a* was mostly active in the mesoderm, with prominent expression in muscles and the intestine, whereas p*ten-1b* was active in the ectoderm, predominantly in neurons, including the soma and axons (Drabikowski et al., 2005). Using specific antibodies against the N-terminal part of TEN-1L, the intracellular domain was detected in the nucleus (Drabikowski et al., 2005). Expression of the *ten-1* transgene that was fused to GFP under the control of p*ten-1b* confirmed epidermal and neuronal expression patterns in the embryonic stage, indicating the potential involvement of TEN-1 in neuronal development (Topf and Chiquet-Ehrismann, 2011).

The depletion of TEN-1 by RNAi results in severe morphological defects. Worms that were injected with RNAi against both transcripts exhibited an increase in embryonic lethality, accompanied by gross defects in hypodermal cell migration. These findings supported the importance of TEN-1 during early worm development. Prominent post-embryonic defects included generally abnormal body morphology, morphological defects in the reproductive system, defects in muscles, and abnormalities in neuronal migration and axonal pathfinding (Drabikowski et al., 2005). A smaller brood size and morphological defects were confirmed in several TEN-1 mutants. Three mutant alleles of *ten-1* have been characterized (*ok641*, *tm651*, *et5*; (Drabikowski et al., 2005; Trzebiatowska et al., 2008; Morck et al., 2010). Ten-1(*ok641*) and ten-1(*tm651*) are null alleles, and ten-1(*et5*) is a hypomorphic allele with a weaker post-embryonic phenotype. Neuronal defects in the TEN-1 mutants were not as penetrant as during RNAi depletion but were predominantly observed in mutant worms that exhibited other morphological defects, including epidermal defects (Drabikowski et al., 2005; Morck et al., 2010). Migration defects were observed in some neurons in otherwise healthy-looking animals, suggesting that TEN-1 function is specifically required for some neurons. However, the migration and pathfinding of neurons also strongly depend on an intact basement membrane. The basement membrane is a specialized extracellular matrix that surrounds most tissues in all Metazoa. TEN-1 is expressed in all major tissues in *C. elegans* and consists of a large extracellular part with several different structural domains, suggesting that it likely interacts with components of the extracellular milieu.

## Genetic Interactions of *Ten-1* in *C. elegans*

Several studies have identified multiple genetic interactions with *ten-1* (Byrne et al., 2007; Trzebiatowska et al., 2008; Morck et al., 2010; Topf and Chiquet-Ehrismann, 2011; Promel et al., 2012). To date, however, none of these interaction partners have been shown biochemically to interact physically with TEN-1. A high-throughput screen identified *glp-1*, a receptor of the NOTCH family, as a *ten-1* interacting partner (Byrne et al., 2007). Glp-1 is essential for the development of worm gonads as well as TEN-1, and the depletion of *glp-1* together with *ten-1* is embryonically lethal. Nevertheless, the functional basis of this interaction remains to be determined. Further attempts to investigate the function of *ten-1* focused on interactions with genes that encode basement membrane receptors and components and genes that are involved in regulating the cytoskeleton, neuronal guidance, and axon outgrowth (Trzebiatowska et al., 2008; Morck et al., 2010; Topf and Chiquet-Ehrismann, 2011). Table 1 presents an overview on these genetic interactions with *ten-1* (The reader is advised to see original publications for further details on the described phenotypes). Based on phenotypical observations of *ten-1* mutant worms that showed the loss of basement membrane integrity, which surrounds the developing gonad in post-embryonic worms, Trzebiatowska et al. (2008) applied a candidate approach and found that *ten-1* genetically interacted with the basement membrane receptor α-integrin and dystroglycan and basement membrane components lamin and nidogen (Trzebiatowska et al., 2008). Double mutants of all four genes together with *ten-1* resulted in a synthetic lethal or sick phenotype that terminated the development of the double-mutant worms during embryogenesis or at an early larval stage. Previous studies in neuroblastoma cells found that the teneurin-2-dependent induction of filopodia formation was more prominent on lamin substrate (Rubin et al., 1999), and chicken teneurin-2 was shown to colocalize with lamin in basement membranes of the optic cup (Tucker et al., 2001). These findings in *C. elegans* suggested that teneurin is a receptor that might act redundantly with integrin or dystroglycan in basement membrane function. The *ten-1* single mutants display pleiotropic phenotype and those seem likely to show genetic interactions with various genes. However, there is specificity of the interaction between *ten-1* and basement membrane components. Mutations in *cle-1* (CoLlagen with endostatin domain 1; vertebrate type XV/XVIII collagen homolog in *C. elegans*) or *unc-52* (perlecan) did not enhance embryonic lethality, larval arrest, or sterility of the *ten-1*(ok641) mutant (Trzebiatowska et al., 2008). Loss-of-function phenotypes of nidogen (*nid-1*), dystroglycan (*dgn-1*), and integrin (*ina-1*) in worms involve defects in the nervous system (Baum and Garriga, 1997; Kang and Kramer, 2000; Kim and Wadsworth, 2000). However, Trzebiatowska et al. (2008) did not investigate neuronal defects in double-mutant worms. Such studies may be difficult because of the early death of such mutant animals. An unbiased genetic screen of *ten-1*-interacting partners identified *phy-1*, a prolyl-4-hydroxylase that is important for the modification of procollagens, which are secreted into the extracellular milieu, including basement membranes. The *ten-1* also genetically interacts with collagen IV (*let-2* in *C. elegans*); (Topf and Chiquet-Ehrismann, 2011). Collagen IV is required for the completion of embryonic development, tissue organization, and structural integrity. Collagen IV is produced in muscle cells, and insufficient maturation results in the intracellular retention and aggregation of procollagen. Consequently, the combined loss of *phy-1* and *ten-1* resulted in deteriorated connections between the epidermis and muscle tissue. *Drosophila* Ten-a protein also localizes to muscle attachment structures (Kenzelmann-Broz et al., 2010), and the mouse teneurin isoform TEN3 (Odz3) colocalizes with collagens I and II (Murakami et al., 2010). Epidermal defects in *ten-1* and *phy-1* double-mutant worms were accompanied by neuronal defects. Further evidence that TEN-1 is involved in neuronal guidance was provided by a candidate approach, in which defects in pharyngeal neurons were quantified, with a focus on M2 neurons (Morck et al., 2010). The loss of *ten-1* together with genes that are involved in M2 cell body positioning and axon outgrowth resulted in more sever defects in M2 neuron. Among the interacting genes are *sax-3* gene and downstream-acting *unc-34* gene, which are involved in multiple aspects of sensory, motor, and interneuron axon guidance.

**Table 1:** Genetic interactions with *ten-1* that shape neuronal networks in *C. elegans*.

Gene/allele	Description	Phenotype single mutant	Phenotype with *ten-1*(ok641)	Human homolog	Reference
*ten-1*(ok641)	Teneurin	Embryonically lethal, larval arrest, sterile, fertile adults 45%	NA	Teneurin 1 Teneurin 2 Teneurin 3 Teneurin 4	Drabikowski et al., 2005
*ina-1*(gm144)	α-integrin subunit	Larval arrest, sterile, fertile adults 36%	Synthetic lethal	ITGA3, ITGA6, ITGA7	Trzebiatowska et al., 2008
*dgn-1*(cg121)	Dystroglycan	sterile	Synthetic lethal	DAG1	
*epi-1*(RNAi)	Lamin α chain	Embryonically lethal, larval arrest, sterile	Synthetic lethal	LAMA3, LAMA5	
*nid-1*(cg119)	Nidogen	Embryonically lethal, larval arrest, fertile adults 88%	Synthetic lethal	NID1, NID2	
*phy-1*(ok162)	Prolyl 4-hydroxylase	Superficially wild-type	Epidermal and muscle defects, axon guidance defects	P4HA1, P4HA2	Topf and Chiquet-Ehrismann, 2011
*let-2*(g37)	Type IV collagen	larval arrest, fertile adults 90%^1^	Larval arrest 50%^1^	COL4A1, COL4A5, COL4A6	
*mig-14*(ga62)	Wnt-secretion factor	Mild neuronal defects (15%)^2^	Axon guidance defects (64%)^2^	WLS	Morck et al., 2010
*sax-3*(ky123)	Receptor of slit-robo pathway	Axon guidance defects (32%)^2^	Synthetic lethal	ROBO1, ROBO2, ROBO3	
*unc-5*(e53)	Netrin receptor	Axon guidance defects (23%)^2^	Axon outgrowth defects (65%)^2^	UNC5	
*unc-34*(e315)	Ena/VASP homolog	Mild neuronal defects (8%)^2^	Synthetic lethal	EVL	
*unc-51*(e369)	Serine/threonine kinase	Mild neuronal defects (17%)^2^	Axon outgrowth defects (50%)^2^	ULK1, ULK2	
*unc-52*(e1421)	Perlecan	Mild neuronal defects (6%)^2^	Axon outgrowth defects (60%)^2^	HSPG2	
*unc-73*(e396)	Guanine nucleotide exchange factor	Axon guidance defects (50%)^2^	Synthetic lethal	KALRN	
*lat-1*(ok1465)	Latrophilin	Embryonically lethal, larval arrest, fertile adults 30%	Developmental arrest	ADGRL	Promel et al., 2012


*^1^Growth temperature of 20°C. ^2^Observed in pharyngeal M2 neuron; percentage in brackets reflects the animals with defects in M2 neuron. Possibly other neuronal defects are here not taken in account. Synthetic lethal, development ends at embryonic or early larval stage. Wnt, wingless/integrated. Ena/VASP, enabled/vasodilator-stimulated phosphoprotein.*

Genetic interaction data have provided strong evidence that teneurin in *C. elegans* is required for the maintenance of basement membrane integrity. Whether this function is based on structural tasks of teneurin that involve the binding of extracellular proteins or teneurin as a receptor that provides guidance for migrating cells remains to be determined. Nevertheless, this ancient function of TEN-1 may have served to organize and connect tissues, thus providing a foundation for development of the worm’s neuronal network.

## Physical Interactions of Teneurins in Other Species

**FIGURE 1:**
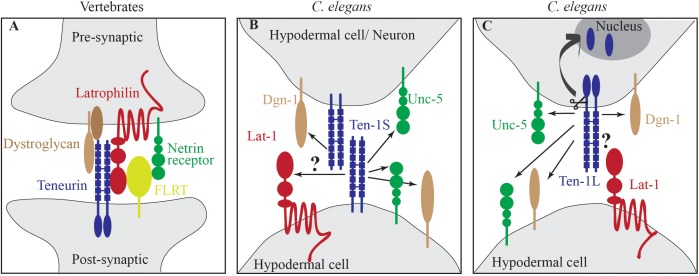
Teneurin-latrophilin interactions. **(A)** In vertebrates, teneurin is a part of protein complexes that connect pre- and postsynaptic parts of neurons. Teneurin physically interacts with latrophilin and also with the basement membrane receptor dystroglycan. Latrophilin is connected to the netrin receptor via FLRT. **(B)** *C. elegans* TEN-1S is expressed in hypodermal cells and neurons and genetically interacts with dystroglycan Dgn-1 and the netrin receptor Unc-5. The latrophilin LAT-1 is expressed in hypodermal cells. Whether TEN-1S and LAT-1 interact is unknown (arrow with question mark). **(C)** *C. elegans* TEN-1L is expressed in many more cells and tissues compared with LAT-1. Thus, *cis* interactions might be possible but have not yet been proven. A presumed interaction between TEN-1L and LAT-1 could trigger the release of the intracellular domain of TEN-1L, initiating cell signaling pathways. FLRT, (fibronectin leucine-rich repeat transmembrane protein), arrows indicate genetic interaction.

Recent biochemical and structural studies in vertebrate systems showed physical interactions between teneurins and other membrane receptors. Particularly interesting is the interaction between teneurin and latrophilin. Latrophilins (LPHN1-3) belong to the adhesion-type G-protein-coupled receptor (GPCR) family. LPHN1 was identified as a receptor for α-latrotoxin, a black widow spider toxin that triggers massive neurotransmitter release from neurons and neuroendocrine cells. In vertebrates, latrophilins interact with FLRTs (fibronectin leucine-rich repeat transmembrane proteins), UNC5 (netrin receptor), neurexins, and teneurins (Davletov et al., 1996; Krasnoperov et al., 1997). Latrophilin, UNC5, and FLRT form a super complex (Lu et al., 2015; Jackson et al., 2016). In neurons, latrophilin is presynaptic and teneurin is postsynaptic, and both proteins engage in *trans* interactions (Figure 1A). A recently published cryo-electron microscopy structure of human latrophilin 1 with teneurin 2 described this interaction in detail (Li et al., 2018).

## *Ten-1* Interaction With *Lat-1*/Latrophilin in *C. elegans*

The *C. elegans* genome contains two latrophilin paralogs, *lat-1* and *lat-2* (Willson et al., 2004; Langenhan et al., 2009). The *lat-1* is expressed in oocytes, early embryonic blastomeres, and precursors of pharyngeal and hypodermal cells. In larvae and adult worms, *lat-1* is expressed in pharynx muscle nerve cells, the gonads, and the vulva. The neuronal and gonadal expression of *lat-1* has only been mentioned and not thoroughly described (Langenhan et al., 2009). Lat-2 is expressed in the pharynx and gland cells of the excretory system. The *lat-1* deletion is embryonically lethal, and the escapees have a smaller brood size because of defects in sperm development. The *lat-2* deletion has no obvious phenotype but enhances the *lat-1*-null phenotype (Langenhan et al., 2009). LAT-1 in *C. elegans* has mostly been studied in the context of early embryogenesis, the alignment of mitotic spindle and division planes, and the establishment of anterior-posterior polarity. Comparing the expression of *lat-1* and *ten-1* is difficult because of insufficient descriptions of *lat-1*::GFP expression patterns. The expression pattern of *lat-1* partially overlaps with *ten-1a* to a small extent in the developing pharynx. LAT-1 expression in dorsal hypodermis during intercalation partially overlaps with *ten-1b* promoter expressing Ten1S version of teneurin (Drabikowski et al., 2005).

In *C. elegans*, *lat-1*, and *ten-1* genetically interact, but the physical interaction has not been demonstrated. In genetic interactions, the alleles displayed non-allelic non-complementation. The loss of any of the alleles of either gene led to developmental defects, and double-heterozygote worms exhibited strong defects in development and fertility (Promel et al., 2012). The authors showed that the *ten-1a* promoter is active in the same half of intercalating hypodermal cells as *lat-1*. Thus, according to these authors, LAT-1 is likely not a receptor for TEN-1L (Figure 1B). Interactions between latrophilin and teneurin in *trans* but not in *cis* have been proven biochemically, microscopically, and structurally in vertebrate systems (Davletov et al., 1996; Krasnoperov et al., 1997). Drabikowski et al. (2005) showed that *ten-1b* promotor-expressing TEN-1S and not *ten-1a*-expressing TEN-1L is expressed in the intercalating dorsal hypodermis in *C. elegans* in both the left and right rows of cells. The approach to obtain transgenic animals undertaken by Promel et al. (2012) expressing *lat-1*::GFP and by Morck et al. (2010) expressing *ten-1a*::GFP promoter fusions often result in random transgene silencing in a subset of cells. Kelly et al. (1997) have shown that simple, highly repetitive extrachromosomal arrays, as in this case used by Morck et al. (2010); Promel et al. (2012), result in transgene silencing. Thus, these expression patterns might reflect only partial expression pattern of LAT-1 and TEN-1 and conclusions drawn from them should be treated with caution. Regardless of whether *lat-1* is expressed in all or only half of intercalating hypodermal cells, TEN-1S appears to be expressed in all intercalating hypodermal cells, thus indicating that in *trans* interactions between LAT-1 and TEN-1S are possible (Figure 1C). TEN-1S protein has a short, 36-amino-acid intracellular domain that does not translocate to the nucleus. Both proteins, TEN-1 and LAT-1, and the processes in which they are involved, are strongly conserved in evolution. Thus, it is highly unlikely that the nature of interactions, in *trans* or in *cis*, between these proteins would not be conserved. The elucidation of endogenous expression patterns of both LAT-1 and TEN-1 in *C. elegans* (e.g., by CRISPR/Cas9 technology) may help resolve these discrepancies.

In *C. elegans*, possible TEN-1 interactions with LAT-1 that are related to neuronal pathfinding and synapse formation await further investigation. In recent years, teneurin research in vertebrates has focused on neuronal function and interactions with latrophilin. Studies of early expression during mouse and chicken embryogenesis have shown that teneurins function not only in neuronal development but also in non-neuronal tissues during the pattern formation of developing limbs (Tucker et al., 2007), somites, and craniofacial mesenchyme (Tucker et al., 2001). Investigations of teneurins in non-neuronal tissues in vertebrates are still incipient but have already opened new avenues of research on both cancer and congenital diseases. Findings in worms may further guide such research.

## Conclusion

Research on teneurin proteins has seen tremendous advances. Teneurins were discovered in 1993 in the labs of Ruth Chiquet-Ehrismann and of Roland Fässler. Since that time, however, the biological role of teneurins in humans has remained elusive. Several excellent studies have been performed in model organisms and cell culture systems, indicating that teneurins play a role as organizers of neuronal networks. Studies of teneurin in *C. elegans* have demonstrated its importance during development. The elucidation of multiple genetic interactions has shown that teneurin is essential for pattern formation, cell migration, and development of the nervous system. The ancestral function of teneurin in the nervous system in *C. elegans* is most pronounced through TEN-1 interactions and the maintenance of basement membrane and tissue integrity. In vertebrates, teneurin function evolved in concert with the multiplication of teneurin genes. Further investigations are required to establish the role of TEN-1 as an organizer of neuronal networks in *C. elegans* and the involvement of LAT-1 in these processes. State-of-the-art genetic tools in worms, combined with detailed descriptions of their development and neuronal connectivity at single-synapse resolution, make this a very promising area of research.

## Author Contributions

UT and KD co-wrote the manuscript and reviewed the references. KD drafted Figure 1.

## Conflict of Interest Statement

The authors declare that the research was conducted in the absence of any commercial or financial relationships that could be construed as a potential conflict of interest.
